# Injuries in girls’ soccer and basketball: a comparison of high schools with and without athletic trainers

**DOI:** 10.1186/s40621-018-0159-6

**Published:** 2018-07-16

**Authors:** Lauren A. Pierpoint, Cynthia R. LaBella, Christy L. Collins, Sarah K. Fields, R. Dawn Comstock

**Affiliations:** 1 Department of Epidemiology, Program for Injury Prevention, Education and Research, University of Colorado Anschutz, Aurora, CO USA; 2 Northwestern University Feinberg School of Medicine and Ann & Robert H. Lurie Children’s Hospital of Chicago, Chicago, USA; 3 Datalys Center for Sports Injury Research and Prevention, Indianapolis, IN USA; 4 Department of Communication, University of Colorado Denver, Denver, CO USA; 5 Department of Pediatrics, University of Colorado School of Medicine, Aurora, CO USA

## Abstract

**Background:**

Sports injuries impose physical and economic burdens on high school athletes, yet only 37% of high schools have access to a fulltime certified athletic trainer (AT). Although intuitively there are multiple benefits of AT coverage, research demonstrating the measurable effect of AT coverage on rates and patterns of injury is limited. Our objective was to investigate the epidemiology of girls’ basketball and soccer injuries in high schools with and without an AT.

**Methods:**

We compared data captured by two similar sports injury surveillance systems during the 2006/07–2008/09 academic years. High School Reporting Information Online (RIO) included a national sample of schools with ATs, and the Sports Injury Surveillance System (SISS) included a sample of Chicago public high schools without ATs.

**Results:**

Overall injury rates were higher in schools without ATs than schools with ATs in girls’ soccer (RR: 1.73, 95% CI: 1.51–2.00) and basketball (RR: 1.22, 95% CI: 1.03–1.45). Recurrent injury rates were even higher in schools without ATs compared to schools with ATs in soccer (RR: 6.00 95% CI: 4.54-7.91) and basketball (RR: 2.99, 95% CI: 2.12–4.14). Conversely, concussion rates were higher in schools with ATs than schools without ATs in soccer (RR: 8.05, 95% CI: 2.00–32.51) and basketball (RR: 4.50, 95% CI: 1.43–14.16). Other injury patterns were similar between the two samples.

**Conclusions:**

This study demonstrated the effectiveness of AT coverage of high school girls’ soccer and basketball, both in reducing overall and recurrent injury rates and in identifying athletes with concussions. Future studies should evaluate the effect of ATs on other high school sports and on youth sports to determine if these findings are generalizable across sports and age groups.

## Background

Sports participation provides numerous benefits for young people, including improved cardiovascular fitness, lower obesity rates, better academic performance, and lower rates of depression (Miller et al. 1999; US Department of Health and Human Services 1996). However, sports participation will always include some inherent risk of injury. In the 2008/09 academic year, an estimated 7.5 million United States (US) high school students participated in sports (National Federation of State High School Associations 2014), with participants in just 9 of the most popular sports incurring an estimated 1.2 million injuries (Comstock et al. 2009). Sports injuries impose physical, emotional and economic burdens on high school athletes and their families, and are one of the leading reasons why young people stop participating in sports and physical activity (Centers for Disease Control and Prevention 2018). The public health challenge facing policy makers, clinicians, coaches, parents and athletes is how to mitigate the injury burden associated with sports to the lowest possible level so that young people may stay involved as a way of incorporating physical activity as part of a healthy lifestyle.

Having certified athletic trainer (ATs) services available to athletes in the high school setting has the potential to reduce the number and severity of sports injuries. The National Athletic Trainer’s Association and the American Medical Association endorse the presence of ATs in secondary schools (National Athletic Trainer’s Association 2018; American Medical Association 2018). ATs are licensed health care professionals who collaborate with physicians on injury prevention, clinical evaluation and diagnosis, immediate and emergency care, treatment and rehabilitation, and professional health and well-being. Examples of ATs’ skills applied at the high school level include but are not limited to (1) developing and implementing emergency action plans and pre-season conditioning programs, (2) advising on safety of equipment, weather, and field conditions, (3) providing first response to and triage of acute injuries, (4) implementing treatment and rehabilitation for injured athletes, and (5) determining readiness for return-to-play after injury (National Athletic Trainer’s Association 2018; Almquist et al. 2004). While application of these skills is intended to reduce injury incidence and severity, the effect of the presence of ATs on sports injury rates and patterns in the high school setting has not previously been directly measured.

The objective of our study was to investigate the epidemiology of girls’ basketball and soccer injuries in high schools with and without an AT over the 2006/07–2008/09 academic years using data from two injury surveillance systems that collected injury information on both sports during the same period of time. To our knowledge, no prior studies have been able to compare injury rates and patterns in schools that have an AT with schools that do not. The specific aims were to 1) calculate injury rates in both samples; 2) describe patterns of injury including injury type, body site injured, mechanism of injury, whether the injury was new or recurrent, and timing of injury during the season in both samples; and 3) compare injury rates and patterns across samples.

## Methods

### High School RIO™

Data from the National High School Sports-Related Injury Surveillance Study, which gathers data via the internet-based surveillance system Reporting Information Online (RIO), were used. During the 2006/07–2008/09 academic years, schools with a National Athletic Trainers’ Association (NATA) affiliated AT with a valid email address were invited to participate. Willing participants were assigned to one of 8 sampling strata based on school size (< 1000 and ≥ 1000) and geographical region (South, Midwest, West, Northeast) (US Census Bureau 2014) with 100 schools randomly selected (12 or 13 from each strata) to obtain a nationally representative sample. If a school withdrew from the study, a replacement school was chosen from the same strata.

Previous studies have described the methodology of this large national high school sports-related injury surveillance system in detail (Rechel et al. 2008; Castile et al. 2012). In brief, ATs logged onto High School RIO weekly to submit injury reports and athlete exposure (AE) information for five boys’ sports (football, soccer, basketball, baseball and wrestling) and four girls’ sports (volleyball, soccer, basketball and softball). Injury reports collected data on the athlete (age, height, weight, etc.), injury (type, body site, mechanism, new versus recurrent, etc.) and event (practice vs. game, etc.). ATs could view, edit and update injury reports throughout the academic year. An AE was defined as one athlete participating in one practice or competition. During the 2006/07 academic year, a reportable injury was defined as one which (1) occurred as a result of participation in an organized practice or competition, (2) required medical attention by an AT or a physician and (3) resulted in restriction of the athlete’s participation for 1 or more days. For the 2007/08–2008/09 academic years, the definition of injury was expanded to capture all concussions, regardless of time loss. For this study, we included only girls’ soccer and basketball injuries captured by High School RIO for comparison with the Sports Injury Surveillance System described below.

### Sports Injury Surveillance System

Sports Injury Surveillance System (SISS) is a secure, internet-based injury surveillance system that was developed for a previous study (LaBella et al. 2011) for which injury and exposure data were collected from 111 girls’ soccer and basketball teams from 36 of the 80 Chicago public high schools offering those two sports. Although the underlying purpose of SISS was a study of knee injuries, data related to all injuries were collected in this study. None of the schools employed ATs. Coaches of enrolled teams were given forms to record which athletes participated in each practice and game and all injuries occurring during a practice or game. Teams at SISS participating schools practiced only three times per week. An injury was defined as one resulting in time loss from a practice/game. An AE was defined as one athlete participating in all or part of a practice or game. Three research assistants (RAs) who were students in medicine, physical therapy, and advanced practice nursing collected the coaches’ forms weekly. RAs interviewed injured athletes and/or their parents following signed parent consent to obtain injury mechanism, injured body part, injury type (e.g. sprain, fracture, contusion, etc.), whether the injury was new or recurrent, type of medical evaluation and treatment, and number of games/practices missed due to injury. Interviewing occurred as soon as possible after injury to minimize recall bias. All interviews were completed by the end of the respective sports season. When available, physician’s notes, imaging study reports, and operative notes were obtained to confirm diagnoses. RAs entered all data into SISS, and could view, edit and update injury reports throughout the study.

### Data analysis

Data were analyzed using SPSS version 19.0 (SPSS, Chicago, IL) and Open Epi v.2.3.1 (CDC). Un-weighted case counts were used throughout for ease of comparison between studies. Rate ratios (RR) and injury proportion ratios (IPR) with 95% confidence intervals (CIs) were calculated, with the lower rate or proportion used as the referent group. CIs not containing 1.00 were considered statistically significant. Sample calculations follow:

$$ \boldsymbol{RR}=\frac{\left(\#\boldsymbol{of}\ \boldsymbol{RIO}\ {\boldsymbol{girls}}^{'}\ \boldsymbol{soccer}\ \boldsymbol{injuries}/\boldsymbol{RIO}\ {\boldsymbol{girls}}^{'}\ \boldsymbol{soccer}\ \boldsymbol{AEs}\right)}{\left(\#\boldsymbol{of}\ \boldsymbol{SISS}\ {\boldsymbol{girls}}^{'}\ \boldsymbol{soccer}\ \boldsymbol{injuries}/\boldsymbol{SISS}\ {\boldsymbol{girls}}^{'}\ \boldsymbol{soccer}\ \boldsymbol{AEs}\right)} $$

$$ \boldsymbol{IPR}=\frac{\left(\#\boldsymbol{of}\ \boldsymbol{RIO}\ {\boldsymbol{girls}}^{'}\ \boldsymbol{soccer}\ \boldsymbol{concussions}/\boldsymbol{total}\#\boldsymbol{RIO}\ {\boldsymbol{girls}}^{'}\ \boldsymbol{soccer}\ \boldsymbol{injuries}\right)}{\left(\#\boldsymbol{of}\ \boldsymbol{SISS}\ {\boldsymbol{girls}}^{'}\ \boldsymbol{soccer}\ \boldsymbol{concussions}/\boldsymbol{total}\#\boldsymbol{SISS}\ {\boldsymbol{girls}}^{'}\ \boldsymbol{soccer}\ \boldsymbol{injuries}\right)} $$

The Colorado Multiple Institutional Review Board and the Children’s Memorial Hospital Institutional Review Board approved this study.

## Results

### Overall injury rates

High school RIO (schools with ATs) captured 1,082,985 AEs and 2186 injury reports in girls’ soccer and girls’ basketball over the 2006/07–2008/09 school years. During this same time period, SISS (schools without ATs) captured 126,266 AEs and 376 injury reports in the same sports (Table 1). The overall injury rates in both sports were significantly higher in schools without ATs than in schools with ATs (girls’ soccer RR = 1.73, 95% CI: 1.51–2.00; girls’ basketball RR = 1.22, 95% CI: 1.03–1.45). In both school settings, each sport had significantly higher injury rates in competition than in practice (schools with ATs: soccer, RR = 4.34, 95% CI: 3.86–4.89 and basketball RR = 3.15, 95% CI: 2.77–3.57 and schools without ATs: soccer, RR = 3.17, 95% CI: 2.43–4.13 and basketball RR = 2.93, 95% CI: 2.11–4.05).

**Table 1:** Comparison of Injury Rates between Schools with (HS RIO) and without (SISS) Athletic Trainers, 2006/07–2008/09

	HS RIO			SISS			RR (95% CI)^b^
	*N*	AE^a^	Rate per 10,000 AE	*N*	AE	Rate per 10,000 AE	
Girls’ Soccer							
All injuries	1203	521,377	23.07	227	56,746	**40.00**	**1.73 (1.51–2.00)**
Competition injuries	778	154,157	50.47	136	18,134	**75.00**	**1.49 (1.24–1.78)**
Practice injuries	425	367,220	11.57	91	38,612	**23.57**	**2.04 (1.62–2.55)**
New injuries	1076	521,377	20.64	144	56,746	**25.38**	**1.23 (1.03–1.46)**
Recurrent injuries^c^	127	521,377	2.44	83	56,746	**14.63**	**6.00 (4.54-7.91)**
Girls’ Basketball							
All injuries	983	561,608	17.50	149	69,520	**21.43**	**1.22 (1.03–1.45)**
Competition injuries	562	167,090	33.63	88	22,921	38.39	1.14 (0.91–1.43)
Practice injuries	421	394,518	10.67	61	46,599	13.09	1.23 (0.94–1.60)
New injuries	845	561,608	15.05	98	69,520	14.10	1.07 (0.87–1.32)
Recurrent injuries^d^	138	561,608	2.46	51	69,520	**7.34**	**2.99 (2.12–4.14)**

^a^*AE* Athlete-exposure, with one athlete participating in one practice or one competition contributing one AE. AEs may be lower in SISS because participating teams practiced only 3 times per week

^b^All RRs calculated with the lower of the two rates as referent group – the group with the significantly higher rate is bolded; CI denotes Confidence Interval. Bolded CIs indicate significance at alpha = 0.05

^c^Recurrent injuries for HS RIO included recurrence (this academic year) (3.0%), recurrence (previous academic year) (7.1%), other (0.2%) and missing (0.2%). Recurrent injuries for SISS included recurrent injury from this season (10.6%), recurrent injury from previous season (10.1%), complication of previous injury (2.2%), recurrence of an injury from another sport (5.7%), recurrence of a non-sport injury (4.8%), complication of a previous injury from another sport (0.4%) and missing (2.6%)

^d^Recurrent injuries for HS RIO included recurrence (this academic year) (4.4%), recurrence (previous academic year) (8.3%), other (0.3%) and missing (1.0%). Recurrent injuries for SISS included recurrent injury from this season (7.4%), recurrent injury from previous season (19.5%), complication of previous injury (0.7%), recurrence of an injury from another sport (2.0%), recurrence of a non-sport injury (2.0%) and missing (2.7%)

### New versus recurrent injuries

Recurrent injury rates in both sports were significantly higher in schools without ATs than schools with ATs, 6.00 times higher for girls’ soccer and 2.99 times higher for girls’ basketball (Table 1). Recurrent injuries also represented a higher proportion of all injuries in schools without ATs than schools with ATs in soccer (IPR: 3.46, 95% CI: 2.54–4.73) and basketball (IPR: 2.44, 95% CI: 1.69–3.51).

### Types of injuries

Strains/sprains were the most common types of injuries in both sports in both school settings (Table 2). Distribution of injury types was similar between the two samples, with the exception of concussions. Concussion rates were significantly higher in schools with ATs than in schools without ATs in soccer (RR: 8.05, 95% CI: 2.00–32.51) and basketball (RR: 4.50, 95% CI: 1.43–14.16) (Table 2). Concussions also represented a higher proportion of all injuries in schools with ATs than schools without ATs in both soccer (IPR: 13.48, 95% CI: 3.32–54.81) and basketball (IPR: 5.24, 95% CI: 1.64–16.72).

**Table 2:** Injuries by Diagnoses in Schools with (HS RIO) and without (SISS) Athletic Trainers, 2006/07–2008/09

	HS RIO		SISS		
	*N* (%)^a^	Rate per 10,000 AE^a^	*N* (%)	Rate per 10,000 AE	RR (95% CI)^b^
Girls’ Soccer					
Strain/sprain	646 (53.7)	12.40	118 (53.9)	**20.79**	**1.67 (1.38–2.04)**
Contusion	144 (12.0)	2.76	42 (19.2)	**7.40**	**2.68 (1.90–3.78)**
Fracture	96 (8.0)	1.84	7 (3.2)	1.23	1.49 (0.69–3.22)
Concussion	148 (12.3)	**2.84**	2 (0.9)	0.35	**8.05 (2.00–32.51)**
Other	169 (14.0)	3.24	50 (22.8)	**8.81**	**2.72 (1.98–3.73)**
Girls’ Basketball					
Strain/sprain	527 (53.8)	9.38	92 (65.2)	**13.23**	**1.41 (1.13–1.76)**
Contusion	69 (7.0)	1.23	8 (5.7)	1.15	1.07 (0.51–2.22)
Fracture	98 (10.0)	1.74	8 (5.7)	1.15	1.52 (0.74–3.12)
Concussion	109 (11.1)	**1.94**	3 (2.1)	0.43	**4.50 (1.43–14.16)**
Other	176 (18.0)	3.13	30 (21.3)	4.32	1.38 (0.93–2.03)

^a^*AE* Athlete-exposure, one athlete participating in one practice or one competition; total N may not add to those found in Table 1 due to missing data

^b^All RRs calculated with the lower of the two rates as referent group – the group with the significantly higher rate is bolded; CI denotes Confidence Interval. Bolded CIs indicate significance at alpha = 0.05

### Body site injured

The ankle was the most commonly injured body site in both sports in both school settings (Table 3). Rates of ankle injuries were higher in schools without ATs in both soccer (RR: 2.65; 95% CI: 2.04–3.43) and basketball (RR: 1.54; 95% CI = 1.05-2.05). Conversely, head/face injuries were sustained at a higher rate in schools with ATs compared to schools without ATs in both soccer (RR: 3.37, 95%CI: 1.48–7.61) and basketball (RR: 3.90; 95%CI: 1.60–9.49).

**Table 3:** Injured Body Sites in Schools with (HS RIO) and without (SISS) Athletic Trainers, 2006/07–2008/09

	HS RIO		SISS		
	*N* (%)^a^	Rate per 10,000 AE^a^	*N* (%)	Rate per 10,000 AE	RR (95% CI)^b^
Girls’ Soccer					
Head/face^c^	186 (15.5)	**3.57**	6 (2.7)	1.06	**3.37 (1.48–7.61)**
Hip/thigh/upper leg	195 (16.2)	3.74	36 (15.9)	**6.34**	**1.70 (1.19–2.42)**
Knee	250 (20.8)	4.79	68 (30.1)	**11.98**	**2.50 (1.91–3.27)**
Lower leg	96 (8.0)	1.84	13 (5.8)	2.29	1.24 (0.70–2.23)
Ankle	253 (21.1)	4.85	73 (32.3)	**12.86**	**2.65 (2.04–3.43)**
Foot	73 (6.1)	1.40	9 (4.0)	1.59	1.13 (0.57–2.26)
Other	148 (12.3)	2.84	21 (9.3)	3.70	1.30 (0.82–2.05)
Girls’ Basketball					
Head/face	157 (16.1)	**2.80**	5 (3.4)	0.72	**3.90 (1.60–9.49)**
Hand/wrist	89 (9.1)	1.58	8 (5.4)	1.14	1.39 (0.67–2.87)
Hip/thigh/upper leg	63 (6.4)	1.11	5 (3.4)	0.72	1.55 (0.62–3.85)
Knee	185 (18.9)	3.29	53 (36.1)	**7.63**	**2.32 (1.71–3.15)**
Ankle	289 (29.6)	5.15	55 (37.4)	**7.91**	**1.54 (1.15–2.05)**
Other	194 (19.8)	3.45	21 (14.3)	3.02	1.14 (0.73–1.79)

^a^AE = Athlete-exposure, one athlete participating in one practice or one competition; total N may not add to those found in Table 1 due to missing data

^b^All RRs calculated with the lower of the two rates as referent group – the group with the significantly higher rate is bolded; CI denotes Confidence Interval. Bolded CIs indicate significance at alpha = 0.05

^c^Head/face includes all injury diagnoses to this body region, including concussions

### Mechanism of injury

Rates of injury were significantly higher in soccer for most mechanism categories in schools without ATs, with the exception of “player-apparatus/equipment” contact. In basketball, rates of injury were significantly higher in schools without ATs only in the “player-surface” and “no contact/overuse” categories (Table 4). In schools with ATs, the most common mechanism of injury in both sports was “player-player contact” (42.5% for soccer and 36.8% for basketball) while in schools without ATs the most common mechanism of injury in both sports was “no contact/overuse” (44.7% for soccer and 36.2% for basketball).

**Table 4:** Mechanism of Injury in Schools with (HS RIO) and without (SISS) Athletic Trainers, 2006/07–2008/09

	HS RIO		SISS		
Mechanism of injury	*N* (%)^a^	Rate per 10,000 AE^a^	*N* (%)	Rate per 10,000 AE	RR (95% CI)^b^
Girls’ Soccer					
Player-player contact	510 (42.5)	9.78	78 (34.5)	**13.75**	**1.41 (1.11–1.78)**
Player-surface contact	168 (14.0)	3.22	31 (13.7)	**5.46**	**1.70 (1.16–2.49)**
Player-apparatus/equipment contact	109 (9.1)	2.09	9 (4.0)	1.59	1.32 (0.67–2.60)
No contact/overuse	389 (32.4)	7.46	101 (44.7)	**17.80**	**2.39 (1.92–2.97)**
Other	24 (2.0)	0.46	7 (3.1)	**1.23**	**2.68 (1.15–6.22)**
Girls’ Basketball					
Player-player contact	362 (36.8)	6.44	47 (31.5)	6.76	1.05 (0.77–1.42)
Player-surface contact	222 (22.6)	3.96	39 (26.2)	**5.61**	**1.42 (1.01–1.99)**
Player-apparatus/equipment contact	62 (6.3)	1.10	7 (4.7)	1.01	1.10 (0.50–2.40)
No contact/overuse	303 (30.8)	5.39	54 (36.2)	**7.77**	**1.44 (1.08–1.92)**
Other	34 (3.5)	0.61	2 (1.3)	0.30	2.10 (0.51–8.76)

^a^AE = Athlete-exposure, with one athlete participating in one practice or one competition contributing one AE, total N may not add to those found in Table 1 due to missing data

^b^All RRs calculated with the lower of the two rates as referent group – the group with the significantly higher rate is bolded; CI denotes Confidence Interval. Bolded CIs indicate significance at alpha = 0.05

### Timing of injury

Preseason injuries represented a higher proportion of all injuries in schools with ATs compared to schools without ATs in soccer (IPR: 5.05, 95% CI: 2.46–10.37) and basketball (IPR: 4.50, 95% CI: 2.08–9.73). In girls’ soccer, schools with ATs had the highest proportion of injuries during the regular season (77.1%) followed by the pre-season (18.0%) and post-season (4.9%), while schools without ATs had the highest proportion during regular season (91.6%), followed by the post-season (4.9%) and pre-season (3.6%). Similarly, in girls’ basketball, schools with ATs had the highest proportion of injuries during regular season (76.0%), followed by preseason (21.3%), and post-season (2.7%), while schools without ATs had the highest proportion during regular season (88.5%), followed by postseason (6.8%) and preseason (4.7%).

## Discussion

This study, the first to directly compare rates and patterns of sports-related injuries between high schools with ATs and those without ATs, provides support for position statements calling for greater AT coverage for high schools. While findings from this small study of high school girls’ soccer and basketball should be confirmed in future studies of larger and broader populations (i.e. larger samples, more sports), the reduced number of all injuries and, more specifically, of recurrent injuries in high schools with ATs indicates that ATs are effective at primary, secondary, and tertiary injury prevention in the high school setting. Explanations of ATs effectiveness include: (1) ATs are able to work with athletic directors, coaches, parents, and athletes to implement evidence-based injury prevention efforts, (2) ATs are able to recognize, triage, and treat/manage injuries, which may reduce severity and/or complications, (3) ATs are able to facilitate rehabilitation programs, and (4) ATs are able to monitor recovery to ensure that injuries are fully rehabilitated before return to play is allowed. Further studies are necessary to determine to what degree each explanation may affect injury rates, but this study is an important first step.

A growing body of research is providing empirical evidence for the impact of the AT on the health and safety of high school athletes. Dompier et al. and Kerr et al. both reported that most high school athletic training room visits were for injuries that did not result in time loss (Kerr et al. 2015; Dompier et al. 2015). In a study of high school football, schools that employed ATs fulltime had higher overall injury rates than schools with part-time ATs, although this association disappeared when restricted to time-loss injuries only (Kerr et al. 2016). These findings indicate that through recognizing and treating a substantial number of non-time-loss injuries, ATs likely prevent at least some from becoming time-loss injuries and furthermore may reduce significant costs to injured athletes’ families who might otherwise seek care in a medical clinic, urgent care center, or emergency room. Results of these studies support our findings that schools without ATs had higher rates of time-loss injuries than schools with ATs, most likely because non-time-loss injuries at schools without ATs may progress to more serious time-loss injuries if unidentified/untreated. Further, we found no contact/overuse injuries occurred at a higher rate in schools without ATs, suggesting that AT coverage provides a protective factor perhaps though injury prevention or early intervention for more minor injury. We were unable to fully evaluate these theories because both the HS RIO and SISS surveillance systems captured only time-loss injuries. Another study comparing emergency preparedness among schools with and without ATs in Oregon showed that schools with ATs were more likely to implement best-practices components of emergency planning (Johnson et al. 2017). This also suggests that ATs play a key role in injury prevention and mitigation.

Injury rates in girls’ soccer and basketball in this study were similar to those of prior studies reporting significantly higher injury rates in competition compared to practice (Rechel et al. 2008; Khodaee et al. 2016). This was true in both school settings. Interestingly despite the consistency regarding competition and practice injury rates, the preseason accounted for a higher proportion of all injuries in schools with ATs compared to schools without ATs for both sports. These findings in schools with ATs are consistent with those in the college setting where ATs are universally present, and preseason accounts for a higher injury rate in practice than post-season (Hootman et al. 2007). One possible explanation is that high school athletes in schools without an AT may end participation after a pre-season injury without the coach realizing an injury was why the athlete left the team. Another possible explanation is that ATs identify and treat more minor, non-time-loss injuries during preseason enabling the athlete to return to play safely and without subsequent injury. Athletes in schools without ATs might play through unreported pre-season injuries or might not report them to the coach until later in the season when the injuries have progressed to time-loss injuries. Additional research is needed to test these hypotheses.

Although rates of concussions were lower in schools without ATs, this is unlikely because fewer concussions are occurring in these schools. Identifying a concussion is a complex process, and evidence suggests that more than 50% of sports-related concussions go unreported (Register-Mihalik et al. 2013; McCrea et al. 2004; Meier et al. 2015). More likely, ATs are better skilled than coaches and athletes in identifying signs and symptoms of concussions and more adamant that athletes suspected of concussion be removed from play until cleared for return by an appropriate health care provider. Having an AT on site at practices and competitions may also allow greater opportunity for athletes to report concussions. Recent research has shown that although high school athletes are generally aware of some signs and symptoms of concussions, they are unable to recognize more subtle or lesser known signs (e.g., irritability, emotional lability) (McCrea et al. 2004). Wallace et al. reported that high school athletes with access to an AT had more concussion knowledge than those without such access and that one of the most common reasons athletes give for not reporting a concussion was not considering the injury serious enough to require medical attention (Wallace et al. 2017). If athletes in schools with ATs under-report due to this belief, most likely athletes in schools without ATs also discount the seriousness of concussions. Since May 2009, all 50 states and the District of Columbia have passed legislation requiring that athletes in school-based sports displaying signs or symptoms of concussion be removed from play and not allowed to return until they have been evaluated by and received written clearance from a qualified health care professional (Safe Kids USA 2018). Many of these laws also require schools to provide coaches, parents and athletes with education about concussion symptoms and the importance of proper management before the start of the sports season (Dompier et al. 2015). Further research should assess legislation effectiveness and the roles that ATs play in facilitating implementation and enforcement of concussion legislation in the high school setting.

Our study has some limitations. There were small differences in data collection methodologies between RIO and SISS. The lower injury rates observed in schools with ATs may be partly because RIO only included injuries that required medical attention from an AT or physician. SISS included all injuries resulting in time loss regardless of whether or not they required medical attention. However, this limitation is probably minor, since the percentage of injuries requiring physician attention were similar for both RIO and SISS, and it is unlikely that injuries captured by RIO causing an athlete to miss a practice or game were not brought to the attention of the school’s AT. A second limitation is that RIO injuries were reported by an AT, while SISS injuries were captured via a multi-factor approach including report by coaches, athletes’ parents and, when available, physicians’ notes. A previous study (Yard et al. 2009) comparing the recording of injury reports between ATs and coaches found only 63.2% agreement on diagnosis. While this is a concern, we believe this limitation’s effect on our results was minimal given overall patterns of injury were similar between the two samples. A third limitation is that while the SISS captured all injuries, not just knee injuries, it was implemented as part of a study on knee injuries. Thus, coaches may have been more focused on reporting knee injuries, and as a result, may have been less aware of other injuries. This would actually be expected to under-estimate the overall injury rate in schools without ATs, which would suggest the true differences in overall injury may be even greater than those reported in this paper, underestimating the effect of ATs in reducing/preventing injuries. A fourth limitation is that RIO included all concussions regardless of time loss for the 2007/08–2008/09 academic years. However, there were only three concussions in RIO among girls’ soccer and basketball players during this time period that were associated with time loss of less than 1 day so this had no measurable effect on our findings. Finally, our study populations were not identical, and likely differed in important ways. For instance, athletes in schools with ATs may be more likely to live in socioeconomically advantaged areas with better access to medical care and other resources, which may improve overall health and fitness, and as a result, decrease risk for injury. This may partly explain the lower injury rates observed in the national sample of schools with ATs compared to the small population of Chicago schools without ATs. However, there are no national surveillance systems currently in place equivalent to High School RIO that capture injury and athlete exposure information from schools without ATs. Because SISS and High School RIO employed similar injury surveillance methodologies during the 2006/07–2008/09 academic years, this study represents the first opportunity to evaluate injury rates and patterns over the same period of time in schools with and without ATs.

Despite these limitations, this study provides important new information regarding the positive impact ATs can have on improving health and safety of high school athletes. The results of this study are significant because currently only 37% of high schools have access to a full-time AT (Pryor et al. 2015). Sports and recreation governing bodies have long worried about litigation over injuries sustained during sport (Fields and Young 2010; Young et al. 2007). In a litigious society, the best way to avoid a lawsuit is to mitigate risk by trying to prevent the injury and by having a rapid, full, and appropriate response to injuries that do occur (Register-Mihalik et al. 2013). The presence of an on-site AT in the high school setting accomplishes both of these goals.

Various medical professional organizations have long supported schools employing certified ATs to improve the health and safety of high school athletes. In 1998, the American Medical Association released a statement supporting the need to create an Athletic Medical Unit “in every school that mounts a sports program” and that a recommended member of the unit be “preferably a NATA BOC certified athletic trainer” (American Medical Association 2018; Lyznicki et al. 1999). The American Academy of Family Physicians also supports this position, encouraging high schools to include an athletic trainer as an integral part of the high school athletic program (American Academy of Family Physicians 2018); the American Academy of Neurology, too, recommends that “a certified athletic trainer should be present at all sporting events, including practices, where athletes are at risk for concussion.” (American Academy of Neurology 2018) The American Academy of Pediatrics recommends efforts should be made by football teams to have athletic trainers at the sidelines during organized football games and practices (Tackling in Youth Football 2015)

## Conclusions

Our study provides data which supports the positive impact of high school ATs in reducing overall and recurrent injury rates and better identifying athletes with concussions. Thus, our findings provide evidence-based support for these position statements calling for greater AT coverage for high school athletes. Future studies should evaluate the effect of ATs on other high school sports and on youth sports to determine if these findings are generalizable across sports and age groups.

## Abbreviations

AE: Athlete exposure
AT: Athletic trainer
CI: Confidence interval
IPR: Injury proportion ratios
NATA: National athletic trainers association
RIO: Reporting information online
RR: Rate ratios
